# The impact of hCG trigger versus dual trigger on reproductive outcomes in elderly infertile women: a retrospective cohort study

**DOI:** 10.3389/fendo.2025.1580610

**Published:** 2025-05-28

**Authors:** Tingting Wang, Jinxin Ren, Zhaokang Qi, Xuanang Li, Shan Xiang, Shuai Zhao, Yi Yu, Fang Lian, Yuewen Zhao

**Affiliations:** ^1^ The First Clinical Medical College, Shandong University of Traditional Chinese Medicine, Jinan, China; ^2^ Department of Reproduction and Genetics, Affiliated Hospital of Shandong University of Traditional Chinese Medicine, Jinan, China; ^3^ CReATe Fertility Centre, Toronto, ON, Canada

**Keywords:** hCG trigger, dual trigger, antagonist protocol, elderly, infertility

## Abstract

**Purpose:**

This study was designed to evaluate the effects of dual trigger (GnRH agonist and hCG) compared with hCG trigger alone on oocyte quality, embryo development, and pregnancy outcomes in elderly women (aged≥35 years) who underwent IVF treatment with an antagonist stimulation protocol, aiming to identify the more optimal triggering strategy.

**Methods:**

This retrospective cohort study analyzed 449 elderly infertile women (≥35 years) who underwent antagonist stimulation protocols, including 236 patients in the hCG trigger group and 213 patients in the dual trigger group. The study compares the age, gravidity, parity, body mass index (BMI),anti-Müllerian hormone (AMH),gonadotropin (Gn) days, Gn dosage, trigger day luteinizing hormone (LH), trigger day estradiol (E2), trigger day progesterone (P), number of follicles ≥14mm on trigger day, number of oocytes retrieved, two pronuclei (2PN) fertilization rate, cleavage-stage embryo number, blastocyst number, embryo implantation rate (IR), clinical pregnancy rate (CPR), live birth rate (LBR), and miscarriage rate between the two groups. Multivariate logistic regression was used to analyze the influencing factors of CPR in patients.

**Results:**

There were no significant differences in baseline and cycle data between the two groups. In terms of oocyte and embryo outcomes, the number of oocytes retrieved (P=0.018), 2PN fertilization rate (P=0.046), and cleavage-stage embryo number (P=0.032) were significantly higher in the dual trigger group than in the hCG trigger group. There was no significant difference in the number of blastocysts obtained in the cycles of the two groups (P=0.689). In terms of pregnancy outcomes, the CPR per embryo transfer (ET) cycle (P=0.010),the CPR per frozen embryo transfer (FET) cycle (P=0.011), total embryo IR (P<0.001), total CPR (P<0.001), CPR per patient (P=0.003), total LBR (P<0.001), and LBR per patient (P=0.001) were all significantly higher in the dual trigger group than in the hCG trigger group. There was no significant difference in the miscarriage rate between the two groups (P=0.841). No cases of ovarian hyperstimulation syndrome (OHSS) occurred in either group.

**Conclusion:**

For elderly women undergoing antagonist stimulation protocols, the use of dual trigger, is more effective than hCG trigger alone in improving oocyte quality, embryo outcomes, and pregnancy outcomes.

## 1 Introduction

In the field of assisted reproductive technology, women aged 35 and above often face clinical challenges such as decreased ovarian reserve function and reduced oocyte quality. The choice of ovulation induction protocol and triggering method has a decisive impact on pregnancy outcomes. In recent years, the dual trigger protocol, as an emerging triggering strategy, has received extensive attention.

Research has demonstrated that the human chorionic gonadotropin (hCG) trigger is highly successful in promoting final oocyte maturation. However, the danger of ovarian hyperstimulation syndrome (OHSS) is greatly increased by its potent and long-lasting effects (which can last up to 48 hours). In contrast, gonadotropin-releasing hormone agonist (GnRH agonist), by promoting the production of endogenous luteinizing hormone (LH) and follicle-stimulating hormone (FSH), can improve oocyte quality. However, its duration of action is relatively short (only 12–36 hours), which may lead to luteal insufficiency and thus affect pregnancy outcomes (1). By temporarily inducing the pituitary to release a substantial amount of gonadotropins, GnRH agonist’s “Flare-up” effect demonstrates that it enhances the synchronous development of follicles and significantly increases the maturation rate of oocytes and the number of high-quality embryos (2, 3). But according to the study by Li X et al. (4), using GnRH agonists and hCG together successfully suppressed the “Flare-up” effect of GnRH agonists, quickly suppress the female gonadal axis, which reduced the risk of OHSS, and increased the success rate of assisted reproductive technology.

The randomized controlled study by Svenstrup L et al. (5) further explored the impact of different triggering protocols on progesterone concentration and the prevalence of OHSS. The findings of the study revealed that compared with the traditional hCG trigger protocol, triggering with GnRH agonist followed by sequential use of hCG support could provide better luteal phase progesterone concentration, but four women still developed OHSS. Therefore, the dual trigger protocol emerged.

The investigation conducted by Chen K et al. (6) found that in women with diminished ovarian reserve, the dual trigger protocol markedly outperformed the hCG single triggering procedure in relation to the amount of oocytes and embryos that were retrieved, and it also dramatically reduced the ET cancelation rate. The dual trigger protocol combines the advantages of hCG and GnRH agonist.

However, in ET cycles, there was no significant difference in the implantation, live birth, and clinical pregnancy rates between the two groups. According to the retrospective cohort study by Dong L et al. (7), there was no significant difference between the dual trigger groups that were and the hCG trigger grouping in terms of the number of oocytes retrieved, the number of available embryos, the plenty of high-quality embryos, the frequency of normal fertilization, the incidence of OHSS, the implantation level, the biochemical pregnancy percentage, the clinical pregnancy level, the ectopic pregnancy pace, the early miscarriage rate, and the live birth speed. Fortunately the miscarriage rate in the dual trigger group was noticeably greater than in the hCG trigger group. Additionally, there was no appreciable difference in the pregnancy rate between hCG trigger and dual trigger, according to Zhang Y et al.’s meta-analysis (8). According to these findings, dual trigger has a similar impact on overall pregnancy outcomes to hCG trigger, albeit having certain advantages in some indicators.

However, the meta-analysis by Beebeejaun Y et al. (9) believed that compared with the traditional hCG trigger protocol, the dual trigger protocol performed better with regard to clinical pregnancy rate (CPR) and live birth rate (LBR). At the same time, the meta-analysis The meta-analysis presented by Hu KL et al. (10) included 1048 participants and concluded that dual trigger had significant advantages over hCG trigger in multiple key indicators. Specifically, dual trigger not only improved the ultimate goal of LBR but also showed better results in early pregnancy indicators (such as clinical pregnancy rate) and oocyte-related indicators (such as the number of oocytes retrieved and embryo quality). These comprehensive results support the use of dual trigger in assisted reproductive technology, especially in scenarios where higher success rates and better treatment outcomes are pursued. However, additional high-quality research is still required to confirm these conclusions because several indicators have a low degree of support.

In studies on specific populations, the study by Zhou C et al. (11) pointed out that in women aged 35 and above, dual trigger did not considerably increase the count of retrieved oocytes, but it did dramatically increase the amount of transferable and top-of-the embryos. The dual trigger group’s live birth and continuing pregnancy rates in frozen embryo transfer(FET) were similar to those of the hCG trigger-only group. Moreover, the retrospective analysis conducted by Tu B et al. (12) included 35 women (each of whom had undergone both dual trigger cycles and hCG trigger cycles), and discovered that dual trigger considerably raised the LBR and CPR in addition to the measure of high-quality and transferable embryos when contrasted with hCG trigger. A prospective investigation also showed that the quantity of oocytes, mature oocytes, and embryo cysts increased when hCG trigger alone was substituted with a dual trigger for ultimate follicle maturation (13). These research results indicate that the dual trigger protocol may have more significant advantages in specific populations, such as elderly women or patients with reduced ovarian reserve.

In order to better understand the application effect of the dual trigger protocol in the population of elderly infertile women and to provide more accurate guidance for clinical practice and achieve the best treatment outcomes, this study retrospectively analyzed the comparison of pregnancy outcomes in elderly infertile women choosing hCG trigger and dual trigger.

## 2 Materials and methods

### 2.1 Study design

**Figure 1:**
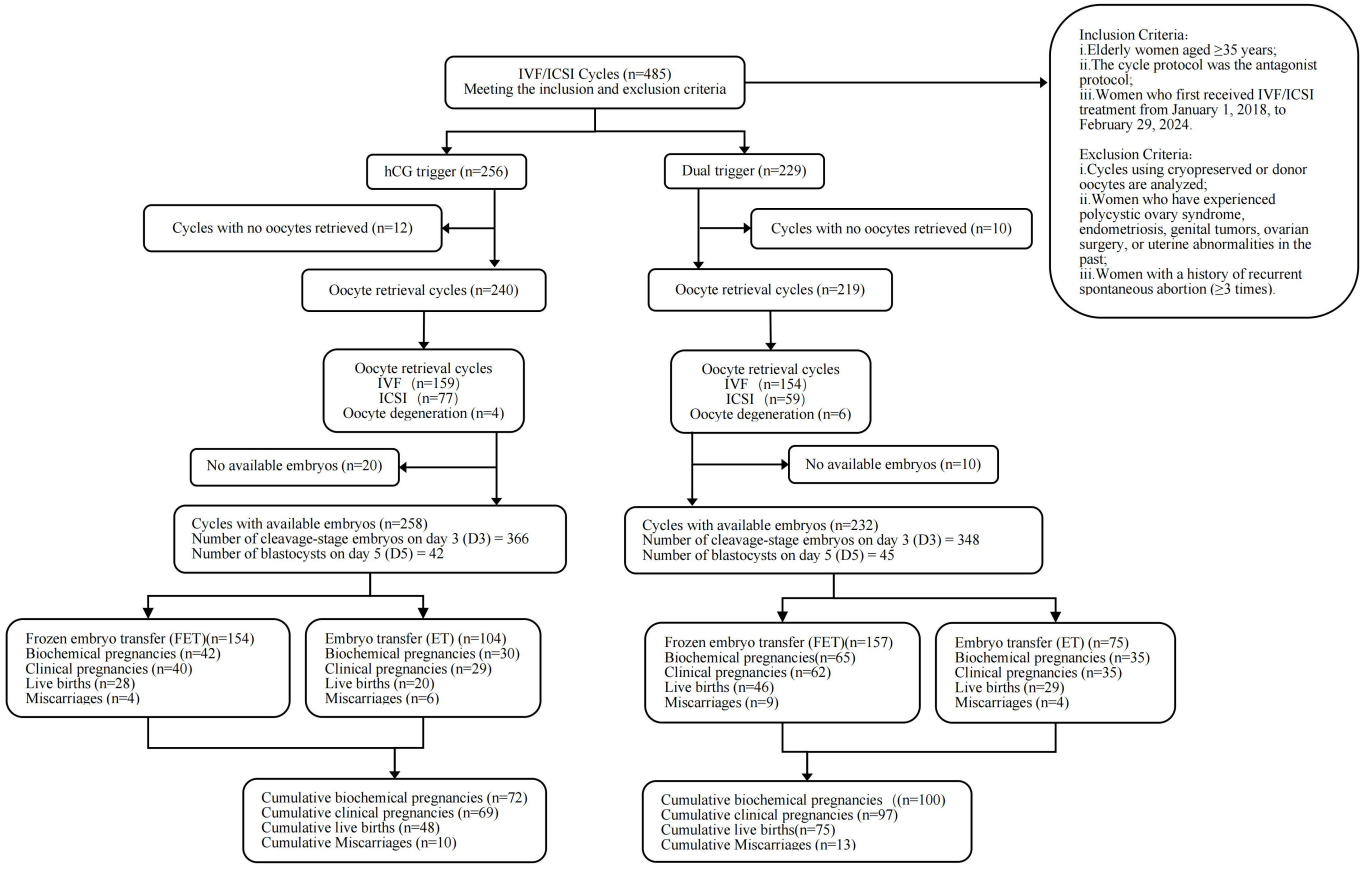
Flow diagram.

A retrospective examination of the cohort has been carried out. From January 1, 2018, to February 29, 2024, we examined the medical records of senior women who initially received treatment with IVF/ICSI cycles with the GnRH antagonist protocol at the Reproductive and Genetic Center of the Affiliated Hospital of Shandong University of Traditional Chinese Medicine. The study was approved by the Ethics Committee of the Reproductive Medicine of the Affiliated Hospital of Shandong University of Traditional Chinese Medicine [Ethical No.2025-009-01-KY]. This study utilized anonymized historical data under a retrospective cohort design, thereby fulfilling criteria for informed consent waiver as endorsed by the ethics committee (**Figure 1**).

### 2.2 Inclusion and exclusion criteria

#### 2.2.1 Inclusion criteria

i. Elderly women aged ≥35 years;
ii. The cycle protocol was the antagonist protocol;
iii. Women who first received IVF/ICSI treatment from January 1, 2018, to February 29, 2024.

#### 2.2.2 Exclusion criteria

i. Cycles using cryopreserved or donor oocytes are analyzed;
ii. Women who have experienced polycystic ovary syndrome, endometriosis, genital tumors, ovarian surgery, or uterine abnormalities in the past;
iii. Women with a history of recurrent spontaneous abortion (≥3 times).

Most of the data came from the electronic medical records of the patients’ IVF/ICSI cycles. To address missing data, we conducted telephone follow - ups and reviewed hospital records at the time of oocyte retrieval. Two groups of 449 IVF/ICSI cycles were created when the inclusion and exclusion criteria were applied: the dual-trigger group (n = 213) and the hCG trigger group (n = 236). The attending physician made the choice between using a dual trigger or just hCG to induce the ultimate oocyte maturation.

### 2.3 Ovarian stimulation protocol

Ovarian stimulation has been performed using the antagonist methodology. On days 2–4 of menstruation, a transvaginal ultrasound examination and baseline hormone measurement were performed. If there were no dominant follicles or functional ovarian cysts, gonadotropin (Gn) (Gonal-f, Merck Serono, Switzerland; or Puregon, MSD, USA; or Livzon, Zhuhai Livzon; or urinary FSH, Zhuhai Livzon) was administered at a dose of 150–300 U/day until the day of triggering. On day 4 of stimulation, follicle development was monitored via transvaginal ultrasound. When follicles ≥12 mm in diameter were detected, gonadotropin-releasing hormone antagonist (GnRH-ant; Cetrotide, Merck Serono, USA) was administered subcutaneously at 0.25 mg/day until the day of triggering.

Based on the triggering mechanism, the study participants were split into two groups: the dual trigger group and the hCG trigger group. Follicle diameter and serum hormone levels were used to determine when to trigger. When two follicles had a diameter ≥17 mm or one follicle had a diameter ≥18 mm, two different triggering regimens were administered based on individual circumstances: (I) Dual trigger regimen: hCG (Livzon, Zhuhai Livzon) 6,000–10,000 U intramuscular injection combined with GnRH agonist (Diphereline, Ferring, Germany) 0.2 mg subcutaneous injection; (II) hCG trigger regimen: hCG 6,000–10,000 U intramuscular injection. In both groups, oocyte retrieval was carried out 34–36 hours following triggering. Beginning on the day of oocyte extraction, 40 mg/d of progesterone injection (Xianju, Taizhou, China) was given as 40 mg im QD to ET patients in order to support the luteal phase. For patients undergoing FET cycles, an incremental substitution protocol was employed for endometrial preparation. The regimen commenced on Day 2–4 of the menstrual cycle with estradiol valerate tablets (Xianju, Taizhou, China) at an initial dose of 4 mg/d. The dose was escalated by 2 mg/d every 4 days: 6 mg/d from Day 6-9, and 8 mg/d from Day 10-13. From Day 14 onwards, the dose was adjusted based on endometrial thickness and hormone levels. Once the endometrial thickness reached the desired state (typically ≥8 mm), the patients proceeded to the progesterone conversion phase. Initiating from the day of endometrial transformation, 40 mg/d of progesterone injections, given as 40 mg im QD, were utilized to sustain the luteal phase in FET patients. Embryo transfer is typically scheduled on Day 3 or Day 5 after the initiation of progesterone administration. Serum β-hCG levels were assessed on day 14 following embryo transfer. Luteal support was continued until the 10th week of gestation while the β-hCG levels were ≥60 mIU/mL, which indicates a positive pregnancy test.

### 2.4 Outcome measures

#### 2.4.1 Baseline data

Age, number of pregnancies, number of deliveries, BMI, AMH, cycle type, number of Gn days, amount of Gn used, and follicular development metrics (LH, E2, P levels, and ≥14 mm follicle count) on trigger day were observed in both groups.

#### 2.4.2 Laboratory data

Oocyte yield, fertilization outcomes (2PN formation), and embryonic development parameters (cleavage-stage embryos and blastocysts) were analyzed. The blastocyst evaluation method employed the Gardner and Schoolcraft grading system (14).

#### 2.4.3 Pregnancy outcome data

The clinical pregnancy rate per ET cycle, the clinical pregnancy rate per FET cycle, the total embryo implantation rate, the total clinical pregnancy rate, the clinical pregnancy rate per patient, the total live birth rate, the live birth rate per patient, and the miscarriage rate were observed.

i. Clinical pregnancy rate per ET cycle: The proportion of clinical pregnancy to ET cycles overall.
ii. Clinical pregnancy rate per FET cycle: The ratio of clinical pregnancies to the total number of FET cycles.
iii. Biochemical pregnancy: 14 days following a successful embryo implantation, a positive pregnancy checkup was obtained(ββtainedyion,g); however, transvaginal ultrasonography did not reveal an intrauterine or extrauterine pregnant sac.
iv. Total embryo implantation rate: The proportion of embryo transfers that are successful to all transfer cycles.
v. Clinical pregnancy: Pregnancy sac detected in the uterine cavity by ultrasound 14 days after a pregnancy test that is positive.
vi. Total clinical pregnancy rate: The overall ratio of transfer cycles to clinical pregnancies.
vii. Clinical pregnancy rate per patient: The percentage of pregnant patients to all patients.
viii. Total live birth rate: The relative proportion of the total number of cycles to the number of live births.
ix. Live birth rate per patient: The proportion of patients who had a successful delivery compared to all patients.
x. Miscarriage: Pregnancy termination with a fetus weighing below 1000 g before the 28th week of development.
xi. Miscarriage rate: The proportion of medical pregnancies to miscarriages.
xii. OHSS Incidence Rate: The proportion of cycles with OHSS occurrence to the total number of oocyte retrieval cycles.

### 2.5 Data management

Two researchers independently screened the medical records of elderly infertile patients satisfying the study requirements at the Reproductive and Genetic Center of Shandong University of Traditional Chinese Medicine from the assisted reproductive technology (ART) system. They entered the data into Excel after extracting the patients’ initial features and pregnancy results. The rules for admission and exclusion were strictly followed in the selection of the study participants. Information was entered twice by two people and checked item by item to ensure the accuracy of the data. In case of any discrepancies, the data were re-searched or followed up and entered again.

### 2.6 Statistical analysis

Statistical analyses were performed using SPSS 27.0. In this study, we employed Multiple Imputation (MI) techniques to handle missing values, thereby ensuring the robustness of the statistical analyses and minimizing the potential biases introduced by missing data. The sample size calculation was based on the primary outcome measure—clinical pregnancy rate. Given an anticipated difference of 15% between the two groups, and considering a significance level (α) of 0.05 and a statistical power of 80%, a minimum of 190 participants were required per group. Accounting for a potential dropout rate of 10%, the final sample size for each group was adjusted to include at least 211 participants, with as many eligible cases as possible being incorporated. Independent sample T-tests were employed for homoscedastic and regularly distributed data, and the data were presented as mean ± standard deviation. We utilized the median (interquartile range) for data exhibiting non-normal distribution. The Kruskal-Wallis test was performed for data that was not normally distributed, while a one-way ANOVA was employed for data that was. Frequencies and percentages were used to express information that is categorical, and the chi-square test or Fisher’s exact test were used to compare groups. Findings are presented as means accompanied by their ± 95% CI. To adjust for potential confounding factors, we employed multivariate logistic regression analysis. By incorporating multiple variables that may influence the clinical pregnancy rate (CPR) into the regression model, we were able to assess the independent effects of each variable while controlling for the others. The variables included in the regression model were age, gravidity, parity, BMI, Gn duration, total Gn dose, hormone levels on the trigger day, number of oocytes retrieved, number of 2PN fertilizations, number of cleavage-stage embryos, and number of blastocysts and a statistically significant P-value was defined as less than 0.05.

## 3 Results

The research enrolled 449 elderly infertile women, with 236 patients in the hCG group and 213 individuals in the dual-trigger group. Data were retrieved from ART cycles from January 1, 2018, to February 29, 2024. Among them, 22 patients had no oocytes (12 in the hCG group and 10 in the dual-trigger group), and 10 patients had oocyte degeneration (4 in the hCG group and 6 in the dual-trigger group). During the transfer cycles involving 449 elderly infertile women, the hCG group accounted for 258 transferable embryo cycles, comprising 154 FET cycles and 104 ET cycles, resulting in the transfer of 366 cleavage-stage embryos and 42 blastocysts. The dual-trigger group had a total of 232 transferable embryo cycles (157 FET cycles and 75 ET cycles), with 348 cleavage-stage embryos and 45 blastocysts transferred.

### 3.1 Baseline and cycle data of patients

No substantial variations existed between the two groups regarding age (39.63 years vs. 39.30 years), gravidity (1.92 vs. 1.67), parity (0.67 vs. 0.71), BMI (24.28 kg/m² vs. 24.28 kg/m²), AMH(1.77ng/ml vs 1.91ng/ml), Gn duration (10.18 days vs. 9.79 days), Gn dosage (2675.64 vs. 2569.25), hCG day LH (3.89 mIU/ml vs. 3.37 mIU/ml), hCG day E2 (2051.67 Pg/ml vs. 2226.11 Pg/ml), hCG day P (1.03 ng/ml vs. 0.98 ng/ml), and the number of follicles ≥14mm on hCG day (8.36 vs. 8.53) (**Tables 1**, **2**).

**Table 1:** Baseline characteristics of patients.

Variable		hCG trigger Group	Dual-Trigger Group	P-value
N (Number of retrieval cycles/patients)		236	213	
Age		39.63 (36.01-43.24)	39.30 (35.67-42.92)	0.327
Parity		1.92 (0.41-3.42)	1.67 (0.22-3.11)	0.080
Gravidity		0.67 (-0.02-1.36)	0.71 (0.01-1.40)	0.505
BMI		24.28 (21.02-27.54)	24.28 (20.62-27.93)	0.989
AMH		1.77 (0.26-3.28)	1.91 (0.41-3.41)	0.368
Type	IVF	159	154	
	ICSI	77	59	

**Table 2:** Stimulation cycle characteristics.

Variable	hCG trigger Group	Dual-Trigger Group	P-value
N	236	213	
Gn duration(days)	10.18 (-4.78-25.15)	9.79 (7.63-11.94)	0.694
Total Gn dose(IU)	2675.64 (1150.59-4200.67)	2569.25 (1781.93-3356.56)	0.347
Trigger day LH(IU/L)	3.89 (0.68-7.09)	3.37 (0.66-6.08)	0.065
Trigger day E2(pg/mL)	2051.67 (610.87-3492.46)	2226.11 (666.23-3785.98)	0.221
Trigger day P(ng/mL)	1.03 (0.40-1.64)	0.98 (0.24-1.70)	0.422
Follicles≥14 mm on trigger day	8.36 (2.38-14.34)	8.53 (1.78-15.27)	0.783

### 3.2 Oocyte and embryo outcomes

Compared to the hCG group, the dual-trigger group exhibited a statistically significant increase in retrieved oocytes (7.16 vs. 8.61, P=0.018), 2PN fertilizations (4.32 vs. 5.14, P=0.046), and cleavage-stage embryos (1.97 vs. 2.35, P=0.032) (**Table 3**). No substantial difference was observed in the quantity of blastocysts acquired between the two groups. (0.57 vs. 0.62, P=0.689) (**Table 3**).

**Table 3:** Embryological outcomes.

Variable	hCG trigger Group	Dual-Trigger Group	P-value
N	236	213	
Oocytes retrieved	7.16 (1.73-12.58)	8.61 (1.18-16.03)	0.018
2PN zygotes	4.32 (0.72-7.91)	5.14 (0.08-10.19)	0.046
Cleavage-stage embryos (D3)	1.97 (0.16-3.77)	2.35 (0.37-4.32)	0.032
Blastocysts (D5)	0.57 (-0.57-1.71)	0.62 (-0.70-1.94)	0.689

### 3.3 Pregnancy outcomes

In ET cycles, a total of 104 cycles were included in the hCG trigger group, and 75 cycles were included in the dual trigger group. The clinical pregnancy rate of ET cycles (27.88% vs. 46.67%, P=0.010),implantation rate (28.85% vs. 46.67%, P=0.014),live birth rate(19.23% vs. 38.67%, P=0.004),in the dual-trigger group were far greater than those in the hCG group. No major variation was noticed in the miscarriage rates among the two groups of pregnant patients (20.69% vs. 11.43%, P=0.310) (**Table 4**).

**Table 4:** Pregnancy and neonatal outcomes (ET).

Variable	hCG trigger Group	Dual-Trigger Group	OR(95%CI)	P-Value
N	104	75		
Clinical pregnancy per ET cycle	29/104 (27.88%)	35/75 (46.67%)	0.442 (0.237-0.825)	0.010
Implantation rate	30/104 (28.85%)	35/75 (46.67%)	0.463 (0.249-0.862)	0.014
Live birth rate	20/104 (19.23%)	29/75 (38.67%)	0.378 (0.193-0.741)	0.004
Miscarriage rate	6/29 (20.69%)	4/35 (11.43%)	2.022 (0.511-7.999)	0.310

In FET cycles, a total of 154 cycles were included in the hCG trigger group, and 157 cycles were included in the dual trigger group. In FET cycles, the hCG trigger group also showed significantly lower implantation rate (27.27% vs. 41.40%, P=0.009), clinical pregnancy rate (25.97% vs. 39.49%, P=0.011) and live birth(18.18% vs. 29.30%, P=0.021).No significant difference in miscarriage rate(10.00% vs. 14.52%, P=0.504) was observed between the two groups (**Table 5**).

**Table 5:** Pregnancy and neonatal outcomes (FET).

Variable	hCG trigger Group	Dual-Trigger Group	OR(95%CI)	P-Value
N	154	157		
Clinical pregnancy per FET cycle	40/154 (25.97%)	62/157 (39.49%)	0.538 (0.332-0.870)	0.011
Implantation rate	42/154 (27.27%)	65/157 (41.40%)	0.531 (0.330-0.854)	0.009
Live birth rate	28/154 (18.18%)	46/157 (29.30%)	0.536 (0.314-0.915)	0.021
Miscarriage rate	4/40 (10.00%)	9/62 (14.52%)	0.654 (0.187-2.287)	0.504

Overall analysis revealed that the hCG trigger group had significantly lower total implantation rate (27.91% vs. 43.10%, P<0.001), clinical pregnancy per patient (27.97% vs. 41.31%, P=0.003), and live birth per patient (19.92% vs. 33.80%, P=0.001). No instances of OHSS Incidence Rate were seen in either cohort (0%) (**Table 6**).

**Table 6:** Pregnancy and neonatal outcomes (total).

Variable	hCG trigger Group	Dual-Trigger Group	OR(95%CI)	P-Value
N	236	213		
Total implantation rate	72/258 (27.91%)	100/232 (43.10%)	0.511 (0.351-0.744)	<0.001
Clinical pregnancy per patient	66/236 (27.97%)	88/213 (41.31%)	0.551 (0.372-0.818)	0.003
Live birth per patient	47/236 (19.92%)	72/213 (33.80%)	0.487 (0.318-0.747)	0.001

### 3.4 Regression analysis

We performed logistic regression analysis (**Table 7**), including different variables related to the CPR. The findings demonstrated that the quantity of blastocysts, age, and cleavage-stage embryos were important predictors of the CPR. Age exhibited a negative correlation with the clinical pregnancy rate (OR=0.85, P<0.001), while the number of cleavage-stage embryos obtained on D3 had a positive correlation with the clinical pregnancy rate (OR=1.6, P<0.001), and the number of obtained blastocysts was positively correlated with the clinical pregnancy rate (OR=1.562, P<0.001). Other variables had no significant correlation with the clinical pregnancy rate.

**Table 7:** Multivariate logistic regression analysis of factors associated with clinical pregnancy rate.

Variable	β-Value	Standard Error	Wald	P-Value	OR(95%CI)
Age	-0.162	0.041	15.531	<0.001	0.85 (0.785-0.922)
Gravidity	0.035	0.1	0.122	0.727	1.036 (0.851-1.26)
Parity	0.233	0.143	2.653	0.103	1.262 (0.954-1.669)
BMI	-0.008	0.036	0.05	0.824	0.992 (0.925-1.064)
Gn duration	-0.023	0.024	0.897	0.344	0.977 (0.931-1.025)
Total Gn dose	0.000	0.000	1.947	0.163	1.000 (1.000-1.001)
Follicles≥14 mm on trigger day	0.005	0.043	0.012	0.914	1.005 (0.923-1.093)
Trigger day LH	0.013	0.042	0.096	0.757	1.013 (0.933-1.101)
Trigger day E2	0.000	0.000	0.069	0.793	1.000 (1.000-1.000)
Trigger day P	0.143	0.193	0.548	0.459	1.153 (0.791-1.683)
Oocytes retrieved	-0.013	0.051	0.061	0.805	0.987 (0.893-1.092)
2PN zygotes	-0.028	0.06	0.214	0.644	0.973 (0.865-1.094)
Cleavage-stage embryos (D3)	0.47	0.094	25.096	<0.001	1.6 (1.331-1.924)
Blastocysts (D5)	0.446	0.106	17.675	<0.001	1.562 (1.269-1.923)
Constant	3.795	1.726	4.836	0.028	44.495

## 4 Discussion

The prevalence of elderly infertile women has risen over the past decade due to postponed childbirth and governmental changes. Enhancing the pregnancy effects for these women has emerged as a prominent area of research. Advanced age is often accompanied by a decline in oocyte quality, and choosing an appropriate triggering protocol for final follicle maturation to obtain more available embryos is crucial for improving the pregnancy outcomes of elderly infertile women.

In previous studies, Yan MH et al. executed a controlled, randomized experiment comparing dual trigger with hCG trigger alone in patients with low oocyte maturity rates among expected normal ovarian responders (NORs). The findings demonstrated that the oocyte maturity rate in the dual trigger group was markedly superior to that in the hCG trigger group, indicating that dual trigger can more effectively promote oocyte maturation. Additionally, the CPR and cumulative LBR in the dual trigger group were also markedly elevated compared to those in the hCG trigger group, further confirming the advantage of dual trigger in improving reproductive outcomes (15). An RCT study by Ali SS et al. (16) also confirmed this view, suggesting that dual trigger can improve oocyte maturity and embryo quality. Moreover, the dual trigger method does not significantly increase the incidence of OHSS in patients (17–20), indicating that this triggering method not only improves the success rate of pregnancy but also demonstrates good safety. Zhou X et al. (17) pointed out that hCG, due to its long half-life and potent luteal support effect, is the main inducer of OHSS. In this regard, GnRH agonists induce a dual peak release of endogenous LH and FSH by activating the pituitary GnRH receptors, mimicking the natural gonadotropin fluctuations (18, 19). Compared with hCG triggering, which only produces an LH effect, the gonadotropin peak induced by GnRH agonist triggering not only has a shorter half-life but also achieves more physiological oocyte maturation through the synergistic effect of FSH. Studies have shown that this dual-trigger mechanism effectively antagonizes the sustained action of hCG by reducing the production of vasoactive substances and accelerating luteolysis (17, 19). Griffin D et al. (20) further confirmed that the GnRHa triggering strategy has become the most effective clinical intervention for reducing the risk of OHSS. Its core mechanism lies in the precise regulation of gonadotropins and the rapid desensitization of subsequent pituitary function, thereby overcoming the limitations of traditional hCG triggering. It may also reduce the physical, time, and economic burdens on patients due to repeated oocyte retrieval cycles, providing a better choice of ovulation induction strategy for patients. Chen CH et al. conducted a systematic review and meta-analysis comparing dual trigger with hCG trigger alone in IVF/ICSI outcomes during GnRH antagonist cycles. They also concluded that the dual trigger protocol shows significant advantages in optimizing IVF/ICSI outcomes and can be considered a better ovulation induction strategy, especially for patients with poor response to traditional hCG trigger (21). Beebeejaun Y included 12 high-quality RCT studies and conducted a meta-analysis involving 1931 patients, suggesting that dual trigger has shown potential advantages and needs further confirmation (9).

Previously, our team conducted a study in which Dong L et al. (7) retrospectively analyzed the impact of combining GnRH agonist with hCG trigger versus using hCG trigger alone on pregnancy outcomes within the antagonist protocol. The findings revealed that dual trigger was somewhat better than hCG trigger alone regarding the quantity of oocytes retrieved and the number of high-quality embryos; nevertheless, the differences lacked statistical significance. A lack of distinction was found in the prevalence of OHSS within the two groups. Thereafter, the normal fertilization rate, IR, BPR, and CPR in the dual trigger group were slightly higher than those in the hCG trigger group, although the LBR was inferior to that of the hCG trigger group, the differences were not statistically noteworthy. However, the miscarriage rate (MR) in the dual trigger group was higher than that in the hCG trigger group, and the difference was statistically significant. A binary logistic regression study, after controlling for confounding variables, identified age as a major risk factor impacting the clinical rate of pregnancy and live births. Since previous studies lacked age-oriented clinical research, we conducted this study with the main purpose of exploring the impact of dual trigger on elderly infertile women.

Unlike other studies, this research found no statistically significant variance in the miscarriage rates between the two groups. while the CPR, LBR, and other outcomes were significant. This compensates for the differences in the populations included in previous studies and the impact of age and other predictive factors.

Research involving both animals and humans has demonstrated that FSH is crucial for ovulation and egg maturation. It can stimulate the swelling of cumulus cells surrounding the oocyte and encourage luteinized granulosa cells to develop LH receptors, thereby increasing the possibility of obtaining more mature second polar bodies (MII) oocytes (22, 23). The traditional method of using hCG for triggering has defects because it lacks FSH receptor activity and cannot fully simulate the physiological mechanism of natural oocyte maturation and ovulation (24). Natural GnRH is a brief decapeptide released by the hypothalamus that stimulates the pituitary gland to secrete LH and FSH. By modifying the amino acids at positions 6 and 10 in the GnRH molecular structure, GnRH agonist can be synthesized, which have significantly higher biological activity than natural GnRH. Unlike hCG, GnRH agonist triggering can simultaneously cause an elevation of both LH and FSH, a process that is closer to the physiological state of natural ovulation (25).

Nonetheless, inducing oocyte maturation only with GnRH agonist could result in luteal insufficiency, thus diminishing the pregnancy rate and elevating the miscarriage rate (26, 27). By simultaneously injecting hCG, the luteolytic effect of GnRH agonist triggering can be effectively counteracted, providing sufficient luteal support and thus significantly increasing the pregnancy rate (28).

In this study, we observed that the transfer of blastocysts is a significant predictor of clinical pregnancy, but there are still some limitations. Several studies have found that single blastocyst transfer (SBT) may increase the risk of singleton preterm birth (29, 30). Wu Y et al. (31) further investigated and found that compared with SBT, double blastocyst transfer (DBT) increases the risk of twin pregnancy and preterm birth by 20.558 and 3.091 times, respectively. In addition, although the live birth rate of high-quality SBT is lower than that of high-quality DBT, it significantly reduces the risk of adverse pregnancy outcomes. They also found that high-quality SBT may increase the risk of monozygotic twins and significantly increase the proportion of male infants. This indicates that how to improve the clinical pregnancy rate while better avoiding risks will be the focus of future research (32).

One limitation of this study is that the patient population only targeted elderly infertile women, neglecting more refined classifications within this group. From the perspective of AMH, elderly infertile women could be divided into patients with low ovarian response, high ovarian response, and normal ovarian response based on different ovarian reserves. If we had made more accurate groupings based on ovarian reserve in our study, it would have allowed for more suitable treatment plans for elderly women in clinical practice. Previously, He FF et al. (33) found in multiple RCT studies (involving a total of 898 patients) and a meta-analysis that as opposed to hCG trigger alone, dual trigger with GnRH agonist combined with hCG substantially augmented the quantity of oocytes retrieved, MII oocytes, embryos, and high-quality embryos among standard responders. However, these metrics largely revealed no discernible variations among low responders, with only an increase in the number of MII oocytes. Therefore, in GnRH antagonist cycles, dual trigger enhances the quality of the embryo and oocyte maturity in normal responders and may be beneficial for the pregnancy rate in low responders. Dual trigger shown notable benefits in promoting enhancement IVF/ICSI results in patients with poor ovarian response, particularly in lowering the likelihood of OHSS while enhancing oocyte maturity and embryo quality, according to Maged AM et al. (34) who echoed this study. For patients with diminished ovarian reserve (DOR), Lin MH et al. (35) observed that dual trigger significantly elevated the LBR in IVF cycles. This strategy improved the overall success rate of IVF by optimizing oocyte maturation and embryo quality. It not only augmented the volume of oocytes retrieved and mature oocytes but also significantly improved the CPR and LBR. Additionally, this combined triggering method also showed an advantage in reducing the risk of OHSS. Therefore, dual trigger can be an effective strategy in IVF treatment for DOR patients, especially for those with poor response to traditional hCG trigger. However, Chern CU et al. (36) reached the opposite conclusion through a retrospective cohort study. The team assessed the IVF/ET outcomes of dual trigger in contrast to hCG trigger alone in elderly patients with DOR. The results showed that dual trigger did not significantly improve the IVF-ET outcomes in DOR patients. Specifically, dual trigger did not significantly increase the oocyte retrieval rate, available embryo rate, or high-quality embryo rate, nor did it significantly elevate the implantation rate (IR) or clinical pregnancy rate (CPR). The immediate and clinical results of IVF-ET treatment did not significantly differ between dual trigger and hCG trigger alone, even after controlling for variables like age, BMI, and ovarian stimulation protocol. Therefore, this study concluded that in elderly patients with DOR, dual trigger failed to achieve significant gains the immediate or clinical outcomes of IVF-ET, indicating that in this specific patient group, dual trigger did not provide a more significant advantage than hCG trigger alone. Future studies of this nature need to provide larger sample sizes for comparison and offer more precise triggering protocols for different ovarian reserves in elderly infertile women.

The lack of a third group, specifically one that was activated only by GnRH agonists, is another study weakness. If we had included a third group in our study, we could have tested whether triggering with GnRH agonist alone or in combination with GnRH agonist and hCG improved the results demonstrated in the study. Although we believed that adding a third study group (patients receiving only GnRH agonist triggering) would have been more comprehensive, we decided to include only two groups in this study—patients administered hCG trigger versus those receiving a combination of hCG and GnRH agonist triggering. Firstly, the center rarely uses the sole GnRH agonist triggering protocol for elderly infertile women. Secondly, after reviewing the relevant literature, we reported that hCG trigger alone and GnRH agonist triggering alone were not significantly distinct in terms of the quantity of oocytes or embryological results. Therefore, we only included the hCG trigger group and the hCG combined with GnRH agonist triggering group in our study.

In summary, compared to using hCG trigger alone, employing a dual trigger approach for final follicular maturation in older infertile women undergoing IVF enhanced the number of oocytes, mature oocytes, and cleavage-stage embryos. It also improved the rate of embryo implantation, clinical pregnancy, and live birth. The increase in the number of cleavage-stage embryos and blastocysts improved the pregnancy outcomes of IVF cycles. However, the limitation of retrospective studies is that there may be biases, further prospective double-blind research is required to make precise and causal inferences, and further explore the impact of factors from both partners on infertility in elderly women.

## Data Availability

The raw data supporting the conclusions of this article will be made available by the authors, without undue reservation.

## Glossary

COH: Controlled Ovarian Hyperstimulation
hCG: Human Chorionic Gonadotropin
FSH: Follicle-Stimulating Hormone
LH: Luteinizing Hormone
GnRH-a: Gonadotropin-Releasing Hormone Agonist
GnRH-ant: Gonadotropin-Releasing Hormone Antagonist
OHSS: Ovarian Hyperstimulation Syndrome
FET: Frozen Embryo Transfer
ET: Embryo Transfer
CPR: Clinical Pregnancy Rate
LBR: Live Birth Rate
IR: Implantation Rate
E2: Estradiol
P: Progesterone
DOR: Diminished Ovarian Reserve

## References

[1] Yan MH Cao JX Hou JW Jiang WJ Wang DD Sun ZG. GnRH Agonist and hCG (Dual Trigger) Versus hCG Trigger for Final Oocyte Maturation in Expected Normal Responders With a High Immature Oocyte Rate: Study Protocol for a Randomized, Superiority, Parallel Group, Controlled Trial. Front Endocrinol (Lausanne). (2022) 13:831859. doi: 10.3389/fendo.2022.831859 35418945 PMC8996168

[2] Lainas TG Sfontouris IA Papanikolaou EG Zorzovilis JZ Petsas GK Lainas GT. Flexible GnRH antagonist versus flare-up GnRH agonist protocol in poor responders treated by IVF: a randomized controlled trial. Hum Reprod. (2008) 23:1355–8. doi: 10.1093/humrep/den107 18403419

[3] Ghaffari F Jahangiri N Madani T Khodabakhshi S Chehrazi M. Randomized controlled trial of gonadotropin-releasing hormone agonist microdose flare-up versus flare-up among poor responders undergoing intracytoplasmic sperm injection. Int J Gynaecol Obstet. (2020) 148:59–64. doi: 10.1002/ijgo.12988 31569274

[4] Li X Kang X Deng Q Cai J Wang Z. Combination of a GnRH agonist with an antagonist prevents flare-up effects and protects primordial ovarian follicles in the rat ovary from cisplatin-induced toxicity: a controlled experimental animal study. Reprod Biol Endocrinol. (2013) 11:16. doi: 10.1186/1477-7827-11-16 23452939 PMC3598983

[5] Svenstrup L Möller S Fedder J Pedersen DE Erb K Andersen CY. Investigation of luteal HCG supplementation in GnRH-agonist-triggered fresh embryo transfer cycles: a randomized controlled trial. Reprod BioMed Online. (2024) 48:103415. doi: 10.1016/j.rbmo.2023.103415 38452605

[6] Chen K Zhang C Chen L Zhao Y Li H. Reproductive outcomes of dual trigger therapy with GnRH agonist and hCG versus hCG trigger in women with diminished ovarian reserve: a retrospective study. Reprod Biol Endocrinol. (2024) 22:35. doi: 10.1186/s12958-024-01211-z 38566172 PMC10985881

[7] Dong L Lian F Wu H Xiang S Li Y Wei C. Reproductive outcomes of dual trigger with combination GnRH agonist and hCG versus trigger with hCG alone in women undergoing IVF/ICSI cycles: a retrospective cohort study with propensity score matching. BMC Pregnancy Childbirth. (2022) 22:583. doi: 10.1186/s12884-022-04899-2 35869444 PMC9308204

[8] Zhang Y Guo X Guo L Chang HM Shu J Leung PCK. Outcomes comparison of IVF/ICSI among different trigger methods for final oocyte maturation: A systematic review and meta-analysis. FASEB J. (2021) 35:e21696. doi: 10.1096/fj.202100406R 34085322

[9] Beebeejaun Y Copeland T Duffy JMN Sarris I Showell M Wang R. Triggering oocyte maturation in *in vitro* fertilization treatment in healthy responders: a systematic review and network meta-analysis. Fertil Steril. (2024) 123(5):812–26. doi: 10.1016/j.fertnstert.2024.11.011 39547644

[10] Hu KL Wang S Ye X Zhang D Hunt S. GnRH agonist and hCG (dual trigger) versus hCG trigger for follicular maturation: a systematic review and meta-analysis of randomized trials. Reprod Biol Endocrinol. (2021) 19:78. doi: 10.1186/s12958-021-00766-5 34059045 PMC8167939

[11] Zhou C Yang X Wang Y Xi J Pan H Wang M. Ovulation triggering with hCG alone, GnRH agonist alone or in combination? A randomized controlled trial in advanced-age women undergoing IVF/ICSI cycles. Hum Reprod. (2022) 37:1795–805. doi: 10.1093/humrep/deac114 35595223

[12] Tu B Zhang H Chen L Yang R Liu P Li R. Co-administration of GnRH-agonist and hCG (double trigger) for final oocyte maturation increases the number of top-quality embryos in patients undergoing IVF/ICSI cycles. J Ovarian Res. (2024) 17:137. doi: 10.1186/s13048-024-01465-6 38961417 PMC11223314

[13] Haas J Bassil R Samara N Zilberberg E Mehta C Orvieto R. GnRH agonist and hCG (dual trigger) versus hCG trigger for final follicular maturation: a double-blinded, randomized controlled study. Hum Reprod. (2020) 35:1648–54. doi: 10.1093/humrep/deaa107 32563188

[14] Schoolcraft WB Gardner DK Lane M Schlenker T Hamilton F Meldrum DR. Blastocyst culture and transfer: analysis of results and parameters affecting outcome in two *in vitro* fertilization programs. Fertil Steril. (1999) 72:604–9. doi: 10.1016/s0015-0282(99)00311-8 10521095

[15] Yan MH Sun ZG Song JY. Dual trigger for final oocyte maturation in expected normal responders with a high immature oocyte rate: a randomized controlled trial. Front Med (Lausanne). (2023) 10:1254982. doi: 10.3389/fmed.2023.1254982 37869157 PMC10585044

[16] Ali SS Elsenosy E Sayed GH Farghaly TA Youssef AA Badran E. Dual trigger using recombinant HCG and gonadotropin-releasing hormone agonist improve oocyte maturity and embryo grading for normal responders in GnRH antagonist cycles: Randomized controlled trial. J Gynecol Obstet Hum Reprod. (2020) 49:101728. doi: 10.1016/j.jogoh.2020.101728 32173633

[17] Zhou X Guo P Chen X Ye D Liu Y Chen S. Comparison of dual trigger with combination GnRH agonist and hCG versus hCG alone trigger of oocyte maturation for normal ovarian responders. Int J Gynaecol Obstet. (2018) 141:327–31. doi: 10.1002/ijgo.12457 29388691

[18] Fabris AM Cruz M Legidos V Iglesias C Muñoz M García-Velasco JA. Dual trigger with gonadotropin-releasing hormone agonist and standard dose human chorionic gonadotropin in patients with a high immature oocyte rate. Reprod Sci. (2017) 24:1221–5. doi: 10.1177/1933719116682873 28715965

[19] Guo D Pang C Wang K. Comparison of pregnancy outcomes in women with normal ovarian response to the gonadotropin-releasing hormone agonist protocol using different trigger methods: a single-center retrospective cohort study based on propensity score matching. Arch Gynecol Obstet. (2024) 309:2153–65. doi: 10.1007/s00404-024-07404-6 38494512

[20] Griffin D Benadiva C Kummer N Budinetz T Nulsen J Engmann L. Dual trigger of oocyte maturation with gonadotropin - releasing hormone agonist and low - dose human chorionic gonadotropin to optimize live birth rates in high responders. Fertil Steril. (2012) 97:1316 –1320. doi: 10.1016/j.fertnstert.2012.03.015 22480822

[21] Chen CH Tzeng CR Wang PH Liu WM Chang HY Chen HH. Dual trigger with GnRH agonist plus hCG versus triggering with hCG alone for IVF/ICSI outcome in GnRH antagonist cycles: a systematic review and meta-analysis. Arch Gynecol Obstet. (2018) 298:17–26. doi: 10.1007/s00404-018-4751-3 29600322

[22] Griffin D Feinn R Engmann L Nulsen J Budinetz T Benadiva C. Dual trigger with gonadotropin-releasing hormone agonist and standard dose human chorionic gonadotropin to improve oocyte maturity rates. Fertil Steril. (2014) 102:405–9. doi: 10.1016/j.fertnstert.2014.04.028 24842671

[23] Humaidan P Kol S Papanikolaou EG. GnRH agonist for triggering of final oocyte maturation: time for a change of practice? Hum Reprod Update. (2011) 17:510–24. doi: 10.1093/humupd/dmr008 21450755

[24] Blumenfeld Z Eckman A. Preservation of fertility and ovarian function and minimization of chemotherapy-induced gonadotoxicity in young women by GnRH-a. J Natl Cancer Inst Monogr. (2005) 34:40–3. doi: 10.1093/jncimonographs/lgi015 15784821

[25] Engmann L Benadiva C Humaidan P. GnRH agonist trigger for the induction of oocyte maturation in GnRH antagonist IVF cycles: a SWOT analysis. Reprod BioMed Online. (2016) 32:274–85. doi: 10.1016/j.rbmo.2015.12.007 26803205

[26] Engmann L Benadiva C. GnRH agonist (buserelin) or HCG for ovulation induction in GnRH antagonist IVF/ICSI cycles: a prospective randomized study. Hum Reprod. (2005) 20:3258–60. doi: 10.1093/humrep/dei190 16246862

[27] Kolibianakis EM Schultze-Mosgau A Schroer A van Steirteghem A Devroey P Diedrich K. A lower ongoing pregnancy rate can be expected when GnRH agonist is used for triggering final oocyte maturation instead of HCG in patients undergoing IVF with GnRH antagonists. Hum Reprod. (2005) 20:2887–92. doi: 10.1093/humrep/dei150 15979994

[28] Shapiro BS Daneshmand ST Garner FC Aguirre M Hudson C. Comparison of “triggers” using leuprolide acetate alone or in combination with low-dose human chorionic gonadotropin. Fertil Steril. (2011) 95:2715–7. doi: 10.1016/j.fertnstert.2011.03.109 21550042

[29] Zhu Q Zhu J Wang Y Wang B Wang N Yin M. Live birth rate and neonatal outcome following cleavage-stage embryo transfer versus blastocyst transfer using the freeze-all strategy. Reprod BioMed Online. (2019) 38:892–900. doi: 10.1016/j.rbmo.2018.12.034 30954432

[30] Ma X Wang J Shi Y Tan J Guan Y Sun Y. Effect of single blastocyst-stage versus single cleavage-stage embryo transfer on cumulative live births in women with good prognosis undergoing *in vitro* fertilization: Multicenter Randomized Controlled Trial. Nat Commun. (2024) 15:7747. doi: 10.1038/s41467-024-52008-y 39237545 PMC11377718

[31] Wu Y Lu X Chen H Fu Y Zhao J. Comparison of frozen-thaw blastocyst transfer strategies in women aged 35–40 years: a retrospective study. Front Endocrinol (Lausanne). (2023) 14:1141605. doi: 10.3389/fendo.2023.1141605 37404307 PMC10315647

[32] Wu Y Lu X Fu Y Zhao J Ma L. Comparison of frozen-thawed embryo transfer strategies for the treatment of infertility in young women: a retrospective study. PeerJ. (2022) 10:e14424. doi: 10.7717/peerj.14424 36452075 PMC9703987

[33] He FF Hu W Yong L Li YM. Triggering of ovulation for GnRH-antagonist cycles in normal and low ovarian responders undergoing IVF/ICSI: A systematic review and meta-analysis of randomized trials. Eur J Obstet Gynecol Reprod Biol. (2023) 289:65–73. doi: 10.1016/j.ejogrb.2023.08.014 37639817

[34] Maged AM Ragab MA Shohayeb A Saber W Ekladious S Hussein EA. Comparative study between single versus dual trigger for poor responders in GnRH-antagonist ICSI cycles: A randomized controlled study. Int J Gynaecol Obstet. (2021) 152:395–400. doi: 10.1002/ijgo.13405 33011968

[35] Lin MH Wu FS Hwu YM Lee RK Li RS Li SH. Dual trigger with gonadotropin releasing hormone agonist and human chorionic gonadotropin significantly improves live birth rate for women with diminished ovarian reserve. Reprod Biol Endocrinol. (2019) 17:7. doi: 10.1186/s12958-018-0451-x 30609935 PMC6320621

[36] Chern CU Li JY Tsui KH Wang PH Wen ZH Lin LT. Dual-trigger improves the outcomes of *in vitro* fertilization cycles in older patients with diminished ovarian reserve: A retrospective cohort study. PloS One. (2020) 15:e0235707. doi: 10.1371/journal.pone.0235707 32628729 PMC7337315

